# Combination of *Astragalus membranaceous* and *Angelica sinensis* Ameliorates Vascular Endothelial Cell Dysfunction by Inhibiting Oxidative Stress

**DOI:** 10.1155/2020/6031782

**Published:** 2020-09-18

**Authors:** Yonghui Yin, Hui Li, Yumin Chen, Ranran Zhu, Li Li, Xinying Zhang, Ji Zhou, Zichen Wang, Xiaoli Li

**Affiliations:** ^1^Department of Endocrinology, Hospital Affiliated to Shandong University of Traditional Chinese Medicine, Jinan 250011, China; ^2^Department of Emergency, Hospital Affiliated to Shandong University of Traditional Chinese Medicine, Jinan 250011, China; ^3^The Medical Record Room, Hospital Affiliated to Shandong University of Traditional Chinese Medicine, Jinan 250011, China; ^4^Shandong University of Traditional Chinese Medicine, Jinan 250011, China; ^5^Department of Editorial, Shandong University of Traditional Chinese Medicine, Jinan 250355, China

## Abstract

Vascular endothelial dysfunction is an essential and early sign of diabetic macroangiopathy, a primary complication of diabetes mellitus. *Astragalus membranaceous*-*Angelica sinensis* is a classic medical combination applied in China to treat diabetes mellitus. The aim of this study was to investigate the effect of the granule form of the extract produced from the dried root of *Astragalus membranaceous* (AM) combination with the granule form of the extract produced from the dried *Angelica sinensis* (AS) on diabetic macroangiopathy and its underlying mechanism. Herein, rats were treated by AM-AS at a ratio of 3 : 2 via intragastric administration. High glucose-induced human umbilical vein vascular endothelial cells (HUVECs) were then treated with drug-containing serum collected from the rats. In high glucose-treated HUVECs, AM-AS combination increased cell viability (*P* < 0.05), decreased the percentage of apoptotic cells (*P* < 0.05) and the expression of the proapoptosis protein caspase 3 (*P* < 0.05), reduced the proportion of cells in the G0/G1 phase (*P* < 0.05), decreased reactive oxygen species level (*P* < 0.05), enhanced cell migration and invasion (*P* < 0.05), and reduced the level of 8-iso-prostaglandin F2alpha. These results indicate that AM-AS combination at the ratio of 3 : 2 ameliorated HUVEC dysfunction by regulating apoptosis, cell migration, and invasion, which might be mediated by their regulatory effect on reactive oxygen species production. The current study provides a theoretical basis for the treatment of diabetic macroangiopathy using AM-AS.

## 1. Introduction

Diabetes mellitus is a prevalent metabolic disease characterized by hyperglycemia that results in high mortality. With improvements in quality of life and increasingly poor dietary habits, the number of diabetes mellitus patients is increasing every year, and it has been identified as a public health issue [1]. The number of adults suffering from diabetes mellitus worldwide has increased from 108 million in 1980 to 422 million in 2014 [2]. Additionally, 84% of diabetes mellitus patients have died in response to complex stroke and cardiovascular disease [3]. Diabetic macroangiopathy is a primary complication of diabetes mellitus and is a main cause of mortality among diabetes mellitus patients [4]. Evidence has demonstrated that the pathogenesis of diabetic macroangiopathy involves angiogenesis [5], vascular and extracellular matrix remodeling [6], and oxidative stress [7]. Although much effort has been dedicated to diabetic macroangiopathy therapy, no effective drug has been identified to moderate the symptoms [8]. Thus, it is imperative to investigate target drugs and clarify their underlying mechanisms.

Diabetes mellitus has been identified as an emaciation-thirst disease based on the concept of Yin deficiency in China [8], and many traditional Chinese medical formulations have been utilized to treat diabetes mellitus [9–11]. However, because of the complicated composition of the ingredients, the mechanism of traditional Chinese formulations in alleviating diabetes mellitus remains ambiguous. *Astragalus membranaceous*, known as Huang Qi in China, is one of most prevalent traditional Chinese herbal medical compounds with known therapeutic effect against diabetes mellitus [12–14]. *Astragalus membranaceous* has also shown great antioxidative and anti-inflammatory effects [15]. Adesso et al. [16] reported that the *Astragalus membranaceous* extract inhibited oxidative stress and inflammation in intestinal epithelial cells through the activation of NF-*κ*B and Nrf2 signaling. *Angelica sinensis*, known as Dang Gui in China, is another popular traditional Chinese medical ingredient, showing multiple pharmacological activities including cardio protection, antiatherosclerosis, and myocardial infarction prevention [17]. *Astragalus membranaceous* and *Angelica sinensis* combination is a classic combination applied medically in China to invigorate qi and promote blood flow [18], having thus becomes an important research topic in the prevention and treatment of diabetic vascular diseases. Our previous study demonstrated that the combination of *Astragalus membranaceous* and *Angelica sinensis* at a ratio of 3 : 2 exhibited the best protective effect against early diabetic nephropathy [19]. We theoretically analyzed the feasibility of the AM-AS combination in diabetic macroangiopathy treatment [20], but this needs to be further verified experimentally.

On this basis, Sprague Dawley rats received intragastric administration of the granule form of the extract produced from the dried root of *Astragalus membranaceous* (AM) and the granule form of the extract produced from the dried *Angelica sinensis* (AS) at a ratio of 3 : 2 for one week and drug-containing serum was obtained. Vascular endothelial cell injury and dysfunction, which can be induced by high glucose-triggered excessive oxidative stress, are an essential and early determinant of diabetic macroangiopathy [21]. Vascular endothelial cells were treated with drug-containing serum to explore the effect of AM-AS combination on diabetic macroangiopathy and its underlying mechanism.

## 2. Materials and Methods

### 2.1. Component Analysis of AM-AS

AM was supplied by Sichuan New Green Pharmaceutical Technology Development Co., LTD. AS was supplied by Beijing Tcmages Pharmaceutical Co., LTD. The AM and AS were diluted in warm water at a ratio of 3 : 2. A 5 *μ*l aliquot of the combination solution was analyzed using ultrahigh-performance liquid chromatography (Nexera UHPLC LC-30A, Shimadzu, Japan) equipped with a BEH C18 column (1.7 *μ*m, 2.1 × 100 mm, Waters, Massachusetts, USA) maintained at 30°C. Water (A) and acetonitrile (B) were used as the mobile phase for gradient elution at a flow rate of 400 *μ*l/min as follows: 0–3.5 min, 5 ⟶ 15% B; 3.5–6 min, 15 ⟶ 30% B; 6–6.5 min, 30% B; 6.5–12 min, 30 ⟶ 70% B; 12–12.5 min, 70% B; 12.5–18 min, 70 ⟶ 100% B; 18–22 min, 100% B. The detector wavelength was set at 280 nm. The peak area and retention time were used to calculate the concentrations of the compounds.

### 2.2. Preparation of Drug-Containing Serum

Specific pathogen-free Sprague Dawley rats, supplied by the Hubei Provincial Center for Disease Control and Prevention, were housed in a standard 12 h/12 h light/dark cycle with water and food ad libitum at 22–25°C. To obtain drug-containing serum, the rats received intragastric administration of AM-AS (3 : 2) at 5.2 g/kg once a day for one week. The clinical dosage of adult is 50 g granules per day (AM: 30 g; AS: 20 g). The dose of rat was calculated according to the guide for dose conversion between animals and humans [22]. Positive control rats received intragastric administration of simvastatin (SIM, MedChemExpress, New Jersey, USA) at 20 mg/kg once a day for one week. Nontreated rats received intragastric administration of an equal volume of distilled water. Blood collected from abdominal aorta and artery were centrifuged at 3000 rpm at 4°C for 10 min. The supernatant was maintained at −20°C for the follow-up study.

### 2.3. Cell Treatment

Human umbilical vein endothelial cells (HUVECs), purchased from Shanghai Institutes for Biological Sciences, Chinese Academy of Science, were maintained in a F12 K medium (Gibco, Gibco BRL, Gaithersburg, MD, USA) contained 0.1 mg/ml heparin (Bioswamp, Wuhan, China) and 0.05 mg/ml endothelial cell growth supplement (ScienCell, California, USA) at 37°C in a humidified atmosphere of 5% CO_2_. Cells in the logarithmic phase were treated with 25 mM glucose (Sigma, MO, USA) to construct the high glucose-induced HUVEC model [23]. Control cells were treated with 5.5 mM glucose [24]. Then, the cells were divided into four groups: control (CON), model (MOD), MOD + AS-AM combination (3 : 2) (MOD + AS-AM), and MOD + SIM. Cells in the CON group were cultured in Dulbecco's modified Eagle medium (DMEM) containing 5.5 mM glucose and 10% serum from nontreated rats. Cells in the MOD, MOD + AS-AM, and MOD + SIM groups were cultured in DMEM containing 25 mM glucose plus 10% serum from nontreated, AS-AM-treated, and SIM-treated rats, respectively.

### 2.4. 3-(4,5-Dimethylthiazol-2-yl)-2,5-diphenyltetrazolium Bromide (MTT) Assay

Cell viability was detected using an MTT assay. Cells in the logarithmic phase were seeded into a 96-well plate (180 *μ*l, 5 × 10^3^ cells/well) and maintained at 37°C with 5% CO_2_ overnight. After the cells were subjected to different treatments for 24, 48, and 72 h, 20 *μ*l of MTT (5 mg/ml, Bioswamp, Wuhan, China) was added to each well for 4 h at 37°C, followed by incubation with 150 *μ*l of dimethyl sulfoxide for 10 min at room temperature. The absorbance of the wells was analyzed using an AMR-100 apparatus (Leica, Wetzlar, Germany) at 490 nm.

### 2.5. Hoechst 33258 Staining

Hoechst 33258 staining was carried out to qualitatively evaluate cell apoptosis. The cells were subjected to different treatments for 24, 48, and 72 h and fixed with 4% paraformaldehyde for 10 min, followed by Hoechst 33258 (Bioswamp) staining for 3 min. Thereafter, the cells were observed under an inverted fluorescence microscope (Leica).

### 2.6. Flow Cytometry

Cell apoptosis, cell cycle progression, and reactive oxygen species (ROS) generation were assessed using flow cytometry after different treatments. To analyze apoptosis after 24 h of treatment, apoptosis was analyzed using the annexin V-fluorescein isothiocyanate (FITC)/propidium iodide (PI) kit (BD, Shanghai, China). 1 × 10^6^ cells were centrifuged twice at 1000 × *g* at 4°C for 5 min and resuspended in 200 *μ*l of binding buffer (Bioswamp), followed by staining with 10 *μ*l of annexin V-FITC and 10 *μ*l of PI in the dark for 30 min at 4°C. After adding 300 *μ*l of binding buffer, the cells were subjected to flow cytometry (ACEA Biosciences, San Diego, CA, USA). To analyze cell cycle progression, 1 × 10^7^ cells were centrifuged at 1000 × *g* at 4°C for 5 min, followed by resuspension in 300 *μ*l of phosphate-buffered saline (Bioswamp) containing 10% fetal bovine serum (FBS, Gibco, Gibco BRL, Gaithersburg, MD, USA) and 700 *μ*l of absolute ethyl alcohol. The cells were then fixed at −20°C for 24 h and centrifuged at 3000 × *g* for 30 s. The cell precipitate was resuspended in 100 *μ*l of 1 mg/ml RNase A (BD) and maintained at 37°C for 30 min to digest intracellular RNA. The cells were then incubated in 400 *μ*l of PI (50 *μ*g/ml) in the dark for 10 min and subjected to flow cytometry (ACEA Biosciences). For ROS level detection, cells were suspended in diluted 2′,7′-dichlorofluorescin diacetate (DCFH-DA) fluoroprobes (10 *μ*mol/l, Bioswamp) at 1 × 10^7^ cells/ml and incubated for 30 min at 37°C with gentle shaking every 4 min. Nonattached DCFH-DA was removed and the cells were subjected to flow cytometry (ACEA Biosciences).

### 2.7. Western Blot

The protein expression of caspase 3 in HUVECs was measured using western blot after 24 h of treatment. Total proteins were extracted from HUVECs using radioimmunoprecipitation assay lysis buffer (Bioswamp), followed by quantification using a bicinchoninic acid kit (Bioswamp) according to the manufacturer's protocol. 20 *μ*g of proteins were separated by 12% sodium dodecyl sulfate-polyacrylamide gel electrophoresis and transferred onto polyvinylidene fluoride membranes (Millipore, MA, USA). The membranes were then blocked with 5% skim milk at 4°C overnight and incubated with primary antibodies against caspase 3 (1 : 1000 dilution, Bioswamp) and *β*-actin (1 : 1000 dilution, Bioswamp) for 1 h at room temperature, followed by incubation in horseradish peroxidase-conjugated goat anti-rabbit IgG secondary antibody (1 : 20000 dilution, Bioswamp) for 1 h at room temperature. The bands were visualized using a Tanon-5200 apparatus (Tanon, Shanghai, China) and relevant band gray values were read using TANON GIS software (Tanon). *β*-Actin served as an internal reference.

### 2.8. Migration and Invasion Assay

Cell migration and invasion were detected using two-chamber Transwell inserts. Before the experiments, cells were starved in serum-free medium for 24 h. 500 *μ*l of treated cells (1 × 10^5^ cells/ml) were resuspended in F12 K medium (Gibco) supplemented with 1% FBS (Gibco) and seeded in the top chamber. The bottom chamber was filled with 750 *μ*l of F12 K medium supplemented with 10% FBS. The inserts for the cell invasion assay were precoated with 80 *μ*l of Matrigel (BD) between the bottom and top chamber. After 24, 48, 72 h of culture at 37°C, the cells were fixed with 4% paraformaldehyde for 10 min at room temperature and stained with 0.5% crystal violet (Bioswamp) for 30 min. Nonmigrating or noninvading cells were removed using cotton swabs. Migrated or invaded cells were counted using an inverted fluorescence microscope (Leica).

### 2.9. Enzyme-Linked Immunosorbent Assay (ELISA)

After 24 h of treatment, the levels of 8-iso-prostaglandin F2alpha (8-iso-PGF2*α*) in HUVECs was measured using the corresponding ELISA kit (HM10023, Bioswamp) following the manufacturer's protocol.

### 2.10. Statistical Analysis

The data are represented as the mean ± standard deviation (SD). Statistical analysis was performed using IBM SPSS statistics 19.0. Differences between more than two groups were analyzed using one-way analysis of variance followed by Tukey. *P* < 0.05 was considered to be statistically significant.

## 3. Results

### 3.1. Components of AM-AS Combination

**Figure 1:**
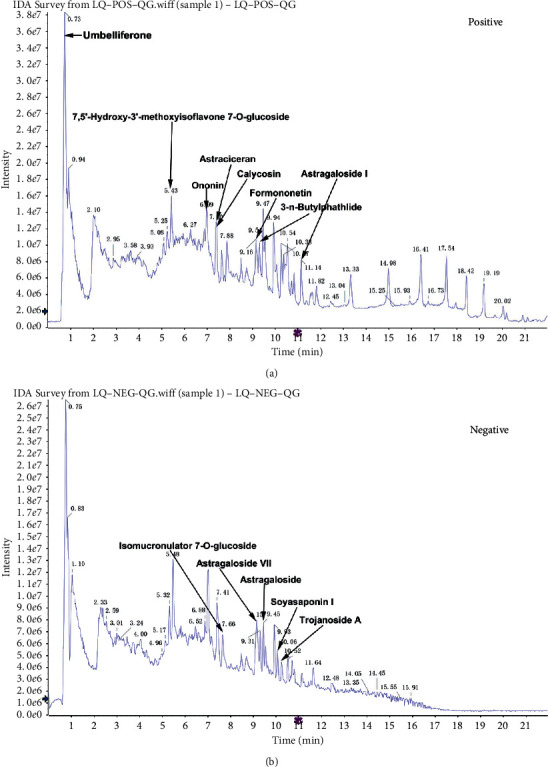
Components of AM-AS. (a) UHPLC-QTOF-MS fingerprint of AM-AS combination in the positive mode; (b) UHPLC-QTOF-MS fingerprint of AM-AS combination in the negative mode.

As shown in Figure 1, the main components of the AM-AS combination are umbelliferose, 7,5′-hydroxy-3′-methoxyisoflavone 7-O-glucoside, ononin, astragaloside I, calycosin, astraciceran, 3-n-butylphathlide, formononetin, astragaloside IV, soyasaponin I, trojanoside A, isomucronulator 7-O-glucoside, and astragaloside VII.

### 3.2. Serum from Rats Treated with AM-AS Combination Recovered the Viability of HUVECs Reduced by High Glucose

**Figure 2:**
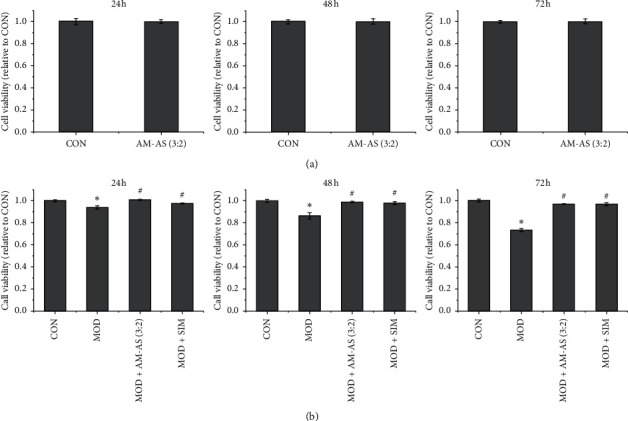
(a) Cell viability of HUVECs in CON and AM-AS (3 : 2) groups by MTT after culture for 24, 48, and 72 h. (b) Cell viability of HUVECs in CON, MOD, MOD + AM-AS (3 : 2), and MOD + SIM groups by MTT after culture for 24, 48, and 72 h. Data represent the mean ± SD (*n* = 3). ^*∗*^*P* < 0.05 vs. CON; ^#^*P* < 0.05 vs. MOD. Cells in the CON group were cultured in DMEM containing 5.5 mM glucose and 10% serum from nontreated rats. Cells in the AM-AS group were cultured in DMEM containing 5.5 mM glucose plus 10% serum from AS-AM-treated rats. Cells in the MOD, MOD + AS-AM, and MOD + SIM groups were cultured in DMEM containing 25 mM glucose plus 10% serum from nontreated, AS-AM-treated, and SIM-treated rats, respectively.

As shown in Figure 2(a), the serum of rats treated with AM-AS showed no effect on the viability of HUVECs, indicating the nontoxic nature of the serum from rats treated with AM-AS combination.  Figure 2(b) demonstrated that high glucose obviously reduced the viability of HUVECs (*P* < 0.05), while the serum of rats treated with AM-AS significantly recovered the viability of HUVECs after it had been decreased by high glucose (*P* < 0.05). The protective effect of serum from AM-AS-treated rats was similar to that of serum from SIM-treated rats.

### 3.3. Serum from Rats Treated with AM-AS Combination Attenuated High Glucose-Induced Apoptosis

**Figure 3:**
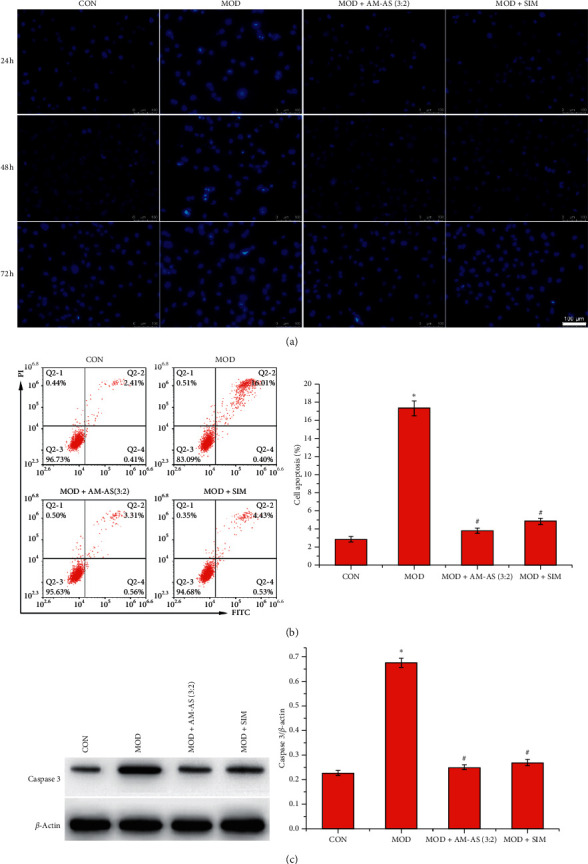
The effect of AM-AS combination on HUVEC apoptosis. (a) Hoechst 33258 staining of HUVECs in CON, MOD, MOD + AM-AS (3 : 2), and MOD + SIM groups after culture for 24, 48, and 72 h. (b) Flow cytometric detection of percentage of apoptotic HUVECs in CON, MOD, MOD + AM-AS (3 : 2), and MOD + SIM groups after culture for 24 h. (c) Western blot of apoptosis-related protein caspase 3 of HUVECs in CON, MOD, MOD + AM-AS (3 : 2), and MOD + SIM groups after culture for 24 h. Data represent the mean ± SD (*n* = 3). ^*∗*^*P* < 0.05 vs. CON; ^#^*P* < 0.05 vs. MOD. Cells in the CON group were cultured in DMEM containing 5.5 mM glucose and 10% serum from nontreated rats. Cells in the MOD, MOD + AS-AM, and MOD + SIM groups were cultured in DMEM containing 25 mM glucose plus 10% serum from nontreated, AS-AM-treated, and SIM-treated rats, respectively.

Hoechst 33258 staining demonstrated that high glucose treatment resulted in apoptosis (brilliant blue), which was rescued by serum from rats treated with AM-AS or SIM (Figure 3(a)). Furthermore, the percentage of apoptotic cells was analyzed by flow cytometry. The results indicated that high glucose remarkably increased apoptosis (*P* < 0.05, Figure 3(b)), whereas serum from rats treated with SIM notably attenuated high glucose-induced apoptosis (*P* < 0.05). The effect of serum from AM-AS-treated rats was similar to that of serum from SIM-treated rats. The results of flow cytometry were consistent with those of Hoechst 33258 staining. In addition, the expression of the apoptosis-related protein caspase 3 was measured, indicating that serum from AM-AS-treated rats downregulated the expression of caspase 3 in HUVECs that was enhanced by high glucose treatment (*P* < 0.05, Figure 3(c)).

### 3.4. Serum from Rats Treated with AM-AS Combination Counteracted High Glucose-Induced G0/G1 Phase Arrest and ROS Production in HUVECs

**Figure 4:**
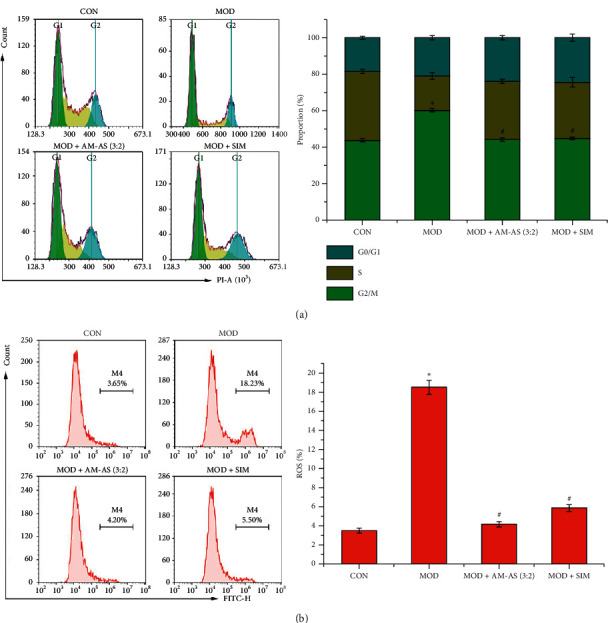
(a) Flow cytometric evaluation of HUVECs cycle progression in CON, MOD, MOD + AM-AS (3 : 2), and MOD + SIM groups. (b) Flow cytometric evaluation of the ROS level of HUVECs in CON, MOD, MOD + AM-AS (3 : 2), and MOD + SIM groups after culture for 24 h. Data represent the mean ± SD (*n* = 3). ^*∗*^*P* < 0.05 v.s. CON; ^#^*P* < 0.05 v.s. MOD. Cells in the CON group were cultured in DMEM containing 5.5 mM glucose and 10% serum from nontreated rats. Cells in the MOD, MOD + AS-AM, and MOD + SIM groups were cultured in DMEM containing 25 mM glucose plus 10% serum from nontreated, AS-AM-treated, and SIM-treated rats, respectively.

Cell cycle and ROS production were detected in HUVECs using flow cytometry. The results demonstrated that high glucose led to G0/G1 phase arrest and enhanced ROS production in HUVECs (*P* < 0.05, Figures 4(a) and 4(b)), which were counteracted by serum from rats treated with AM-AS or SIM (*P* < 0.05, Figures 4(a) and 4(b)).

### 3.5. Serum from Rats Treated with AM-AS Combination Enhanced Dysfunction-Impaired HUVEC Migration and Invasion

**Figure 5:**
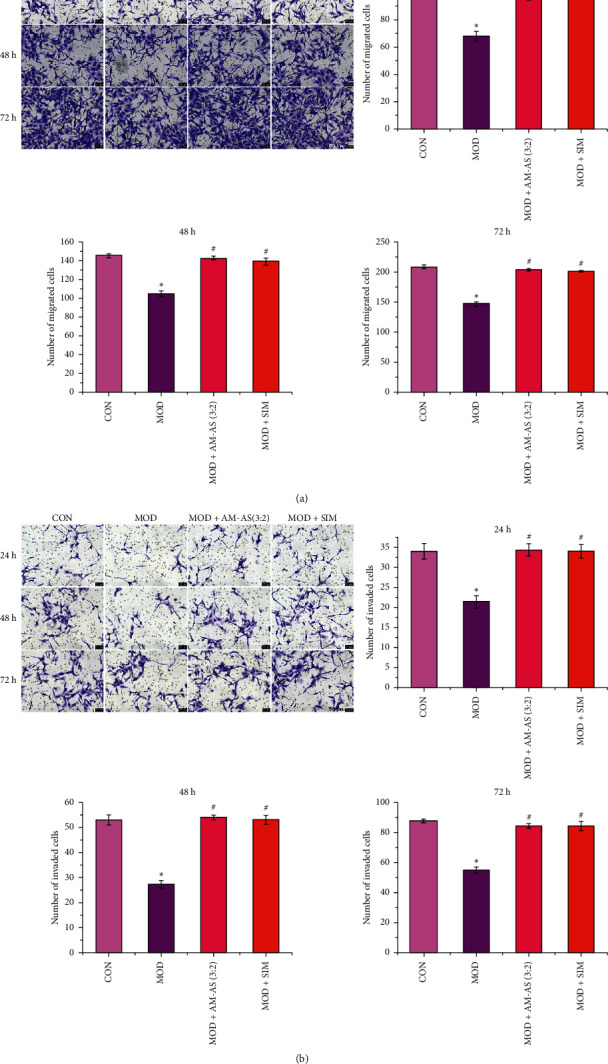
Evaluation of HUVECs (a) migration and (b) invasion in CON, MOD, MOD + AM-AS (3 : 2), and MOD + SIM groups after culture for 24, 48, and 72 h. Data represent the mean ± SD (*n* = 3). Scale bar = 50 *μ*m. ^*∗*^*P* < 0.05 vs. CON; ^#^*P* < 0.05 vs. MOD. Cells in the CON group were cultured in DMEM containing 5.5 mM glucose and 10% serum from nontreated rats. Cells in the MOD, MOD + AS-AM, and MOD + SIM groups were cultured in DMEM containing 25 mM glucose plus 10% serum from nontreated, AS-AM-treated, and SIM-treated rats, respectively.

As shown in Figures 5(a) and 5(b), high glucose impeded the migration and invasion of HUVECs, which was demonstrated by the decrease in the number of migrated and invaded cells (*P* < 0.05). Compared to the cells in the MOD group, the number of migrated and invaded cells in the MOD + AM-AS group and MOD + SIM group were significantly increased (*P* < 0.05).

### 3.6. Serum from Rats Treated with AM-AS Combination Counteracted the Increase in 8-Iso-PGF2*α* Level Induced by High Glucose

**Figure 6:**
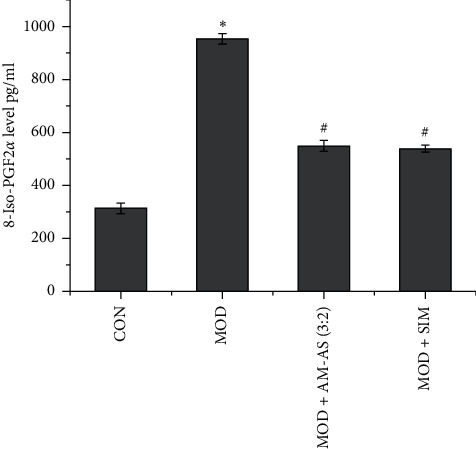
ELISA of 8-iso-PGF2*α* level of HUVECs in CON, MOD, MOD + AM-AS (3 : 2), and MOD + SIM groups after culture for 24 h. Data represent the mean ± SD (*n* = 3). ^*∗*^*P* < 0.05 vs. CON; ^#^*P* < 0.05 vs. MOD. Cells in the CON group were cultured in DMEM containing 5.5 mM glucose and 10% serum from nontreated rats. Cells in the MOD, MOD + AS-AM, and MOD + SIM groups were cultured in DMEM containing 25 mM glucose plus 10% serum from nontreated, AS-AM-treated, and SIM-treated rats, respectively.

Compared to the CON group, the level of 8-iso-PGF2*α* in the MOD group was increased (Figure 6, *P* < 0.05). Compared to the MOD group, the level of 8-iso-PGF2*α* in the MOD + AM-AS and MOD + SIM groups were decreased (Figure 6, *P* < 0.05).

## 4. Discussion

The pathogenesis of diabetes mellitus-induced vascular dysfunction involves dysregulated revascularization or damaged function associated with vascular permeability and homeostasis maintenance in cells such as endothelial cells, stromal cells, inflammatory cells, smooth muscle cells, and pericytes [25, 26]. Vascular endothelial dysfunction induced by excessive oxidative stress is an essential and early determinant of diabetic macroangiopathy [21]. Hyperglycemia is the key pathogeny that drives the development of diabetic vascular complications towards macroangiopathy. High glucose induces oxidative stress, thereby inducing inflammation and cytotoxicity and leading to diabetic macroangiopathy [27, 28]. The underlying mechanism is associated with ROS production during autooxidation of monosaccharides and advanced glycation endproducts, resulting in direct toxicity in the cardiovascular system [29, 30]. Thus, suppressing high glucose-triggered ROS production might be an effective method of treating diabetic macroangiopathy. Abnormal ROS production results in the damage of lipids, DNA, and proteins [31], resulting in cellular dysfunction such as inhibition of cell proliferation [32], migration, and invasion [33] and increased apoptosis [34]. Previous studies have shown that high glucose induced apoptosis in retinal capillary endothelial cells by enhancing ROS production, which was attenuated after the inactivation of ROS-related pathways [35]. In addition, high glucose inhibited HUVEC proliferation and migration [36]. These findings are in accordance with our present work, showing the high glucose-induced ROS production and increased the levels of 8-iso-PGF2*α*, a reliable indicator of ROS production [37]. Our present work also demonstrated that high glucose inhibited HUVEC proliferation, migration, and invasion and enhanced apoptosis.

High glucose-injured HUVECs were treated with AM-AS combination, which reduced ROS generation and alleviated high glucose-triggered HUVEC dysfunction. Component analysis showed that AM-AS contains umbelliferone, astragaloside IV, calycosin, and formononetin, which are associated with diabetes mellitus. Umbelliferone was previously reported to show protective effects against diabetic liver injury by inhibiting inflammatory response and oxidative stress [38]. Astragaloside IV is a dominant active ingredient of AM, and its protective function against diabetes mellitus has been reported [39]. Additionally, astragaloside IV ameliorated vascular endothelial dysfunction through oxidative stress inhibition and calpain-1 activation [40]. Calycosin alleviated diabetes mellitus-induced renal inflammation and cognitive impairment by regulating the NF-*κ*B pathway [41] and reducing oxidative stress-mediated PI3K/Akt/GSK-3*β* signaling [42], respectively. Formononetin showed a protective effect against cognitive impairment in streptozotocin-induced diabetic mice [43] and ameliorated endothelial dysfunction induced by high glucose by inactivating the JAK/STAT pathway [43].

## 5. Conclusion

Collectively, this work demonstrated that AM-AS combination (3 : 2) might ameliorate high glucose-induced HUVEC dysfunction (proliferation, apoptosis, migration, and invasion) by inhibiting oxidative stress (ROS production) through its active ingredients, including umbelliferone, astragaloside IV, calycosin, and formononetin. This work reveals that AM-AS combination (3 : 2) could act as a target drug in the therapy of high glucose-induced diabetes mellitus and complications towards macroangiopathy. However, the underlying specific molecular mechanism is obscured and will be elucidated in follow-up studies.

## Data Availability

The data used to support the findings of this study are included within the article.
